# Surface Characteristics of Machined Polystyrene with 3D Printed Thermoplastic Tool

**DOI:** 10.3390/ma13122729

**Published:** 2020-06-16

**Authors:** Kamalpreet Sandhu, Gurminder Singh, Sunpreet Singh, Raman Kumar, Chander Prakash, Seeram Ramakrishna, Grzegorz Królczyk, Catalin I. Pruncu

**Affiliations:** 1 Department of Product and Industrial Design, Lovely Professional University, Phagwara 144411, India; Kamalpreet.23686@lpu.co.in; 2 Science et Ingénierie des Matériaux et Procédés (SIMAP), Universite Grenoble Alps, 38400 Grenoble, France; Gurminder.singh@grenoble-inp.fr; 3 Mechanical Engineering, National University of Singapore, Singapore 117584, Singapore; snprt.singh@gmail.com (S.S.); seeram@nus.edu.sg (S.R.); 4 Department of Mechanical Engineering, Guru Nanak Dev Engineering College, Ludhiana 141006, India; sehgal91@gndec.ac.in; 5 School of Mechanical Engineering, Lovely Professional University, Phagwara 144411, India; chander.21503@lpu.co.in; 6 School of Mechanical Engineering, Opole University, 45-758 Opole, Poland; g.krolczyk@po.opole.pl; 7 Department of Mechanical Engineering, Imperial College London, Exhibition Rd., London SW7 2AZ, UK; 8 Department of Mechanical Engineering, School of Engineering, University of Birmingham, Birmingham B15 2TT, UK

**Keywords:** three-dimensional printing, fused deposition modelling, dimension accuracy, surface roughness, milling, expandable polystyrene, thermoplastic tool

## Abstract

An effort is made in this work to appraise the surface characteristics of machined expandable polystyrene (EPS) with a novel 3D printed thermoplastic acrylonitrile-butadiene-styrene (ABS) tool. Linear grooves on EPS were made on a vertical milling machine that was modified to conduct experiments in the laboratory. The tests were designed as per the Taguchi L9 based factorial design of experimentation while varying process parameters such as depth of cut, spindle speed, and feed rate. The machining responses dimensional accuracy and surface roughness of the machined grooves were studied. Furthermore, the surface topography of the machined specimens was considered to investigate the mechanism of material removal in response to the processing conditions. Moreover, mathematical models developed for the prediction of the output responses showed a significant correlation with the experimental results. The results of the statistical study indicate that the surface roughness is influenced by the spindle speed and dimensional accuracy by the depth-of-cut. Overall, the findings of the experimental work advocated the feasibility of 3D printed thermoplastic tools for machining soft polymeric materials. It can become a useful alternative for mass and batch production.

## 1. Introduction

In today’s competitive world, industries are rigorously spotlighting on essential aspects such as time, quality, and cost of products to manage the immense pressure [1]. As the need for sustainable manufacturing increases [2], researchers have started to explore the various processes which can deliver the desired outcomes as compared to traditional production activities like machining or injection moulding, while taking care of environmental perspectives [3,4]. However, the three-dimensional (3D) printing methods are often explicit with their specific feedstock materials as compared to conventional manufacturing [5]. Indeed, in the 3D printing technologies, a robust computerized model is used as input design, which results in the saving of material [6]. Since the invention of 3D printing technologies in the 1980s, the pace has gradually shifted from prototyping to rapid manufacturing while growing its customization level [7]. Moreover, in today’s manufacturing scenario where the design of the industrial products changes very often, owing to the change in the lifestyle of the customers, 3D printing technology is the only available option that can cope with sudden design changes quickly and cost-effectively [8]. Furthermore, the continuous technological innovations in 3D printing technologies have set a new paradigm wherein the printed products are being used for the end-user functional and non-functional applications [9,10]. It was observed that amongst different types of 3D printing technologies, fused deposition modelling (FDM) served most of such applications [11,12]. FDM is a well-established technology, the working principle, structural schematic, and input process variables are already documented in the literature [13,14,15]. It was observed that the FDM system is straightforward, relatively cost-effective in terms of equipment and materials, demands less maintenance, and is widely suitable for most of the engineering polymers and their composites [16,17,18]. As a consequence, FDM is commonly used for the manufacture of experimental models, prototypes, and engineering components where the prints produced are exclusively the task of input process parameters [19,20]. However, on the downside, this system suffers from numerous demerit characteristics, for example, low production speed, poor surface quality, etc. [21].

Acrylonitrile-butadiene-styrene (ABS) is one of the mainly preferred commercial feedstock material; however, alternative materials could be easily fabricated [22,23]. The literature reveals that researchers have developed FDM’s in-house feedstock by utilizing a range of polymer matrices [24,25,26] and reinforcements [27,28,29] to meet sophisticated requests of highly demanding end-user applications [30,31]. Furthermore, 3D printed tools are the amongst the mainly chosen industrial applications of FDM, as illustrated by Hierl et al. [32], Kumta et al. [33], Ciocca et al. [34], and Masood and Song [35]. 3D printed tools are also used for developing customized surgical guides and templates [36,37]. Additionally, the products of FDM are acceptable as sacrificial patterns for various types of casting operations [38]. In another novel application of FDM, researchers suggested that the technology has the efficiency for producing tools for grinding operations [39], bioengineering tools [40,41], automotive parts [42], load-bearing apparatus [43], drug-bearing gadgets [44], and sensors/actuators [45]. In [46,47], the authors have studied the efficiency of FDM based tools for sheet metal forming operations.

Haeberle and Desai [47] compared the performance of traditional CNC and polystyrene based thermoform tooling and found that the latter are cost-effective, with comparable quality, quicker in cutting progress time, and capable of producing yields 50% less than the former. Further, ABS was identified as one of the most economical choices to obtain customized tools for a variety of end-user applications [47]. In [48], it was highlighted that the 3D printed tooling provides an excellent alternative to conventional metallic counter parts. Masood and Song [35] developed iron particles in nylon type matrix inserts through 3D printing and obtained excellent tensile properties. Table 1 lists the various research efforts made on the machining of soft polymers.

From the literature review, it was found that minimal studies are available for producing FDM-based machining tools for soft polymeric materials, for example, polystyrene. The present study investigates the efficiency of the FDM-based ABS tool for machining expandable polystyrene (EPS). Further, ABS is a potential candidate for developing customized tooling, through 3D printing, owing to its high tensile strength, desirable hardness, wear resistance, and corrosion resistance. Linear grooves have been made on the EPS surface at variable combinations of input process variables. The effect of spindle speed, feed rate, and depth-of-cut were studied on the finally obtained samples with measured surface roughness and dimensional accuracy. The test runs were appraised through the Taguchi L9 factorial design of experimentation.

## 2. Materials and Methods 

**Figure 1:**
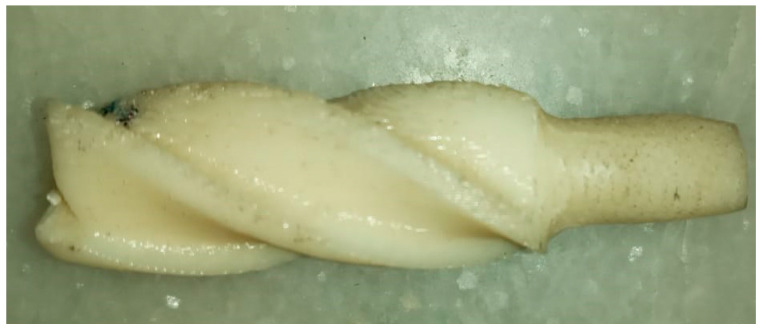
As-printed acrylonitrile-butadiene-styrene (ABS) end-mill tool.

The EPS was utilized as workpiece material in the current study, and Thermo Packers, Jalandhar, India supplied it. As per the supplier’s data-sheet, the EPS has a density of 30–45 kg/m^3^, thermal conductivity of 0.034 W/mK, molecular weight of 224 × 103, polydispersity index > 2.3 Mw/Mn, density of 1.01 g/cm^3^, and MFI of 10.2 g/10 min. The key reasons behind the selection of EPS in this study were: (i) it has widespread utility as a packaging material, (ii) it demands post-operations to engrave fine details through machining, (iii) it is softer and presents potential applications to use 3D printed polymeric tools while post-processing, and (iv) the presence of the voids in the EPS matrix challenges the post-processing. The CREO 4.0 software was utilized to design the end-mill cutting tool; outside φ of 15 mm; the number of flutes/teeth at 4; thickness of the tooth of 1 mm; flute length of 45 mm; overall length of 50 mm; and helix angle of 20°. The standard tessellation language (STL) format was used to slice using Slicer3r and STL of layer thickness 0.254 mm is attained from the CAD model. Every test model was retained to a precision of chord height of 0.0593 and an angle conversation format of 0°. After this, the tool was printed with commercially available ABS feedstock (φ of 1.75 mm) using the FDM system (make: Divide by Zero, Pune, India). The part infill density of 100% was utilized at 35 mm/min of speed to print the end-mill tool with orientation parallel to the bed at a raster angle of ±45°. These printing parameters have been obtained from [49,50]; these maintained the minimal deviations of dimensions of the constructed tool in comparison to the CAD model, as well as producing superior surface quality. The width of 14.998 mm was obtained for the printed end-mill tool and is utilized for the next machining operations, as shown in Figure 1.

**Figure 2:**
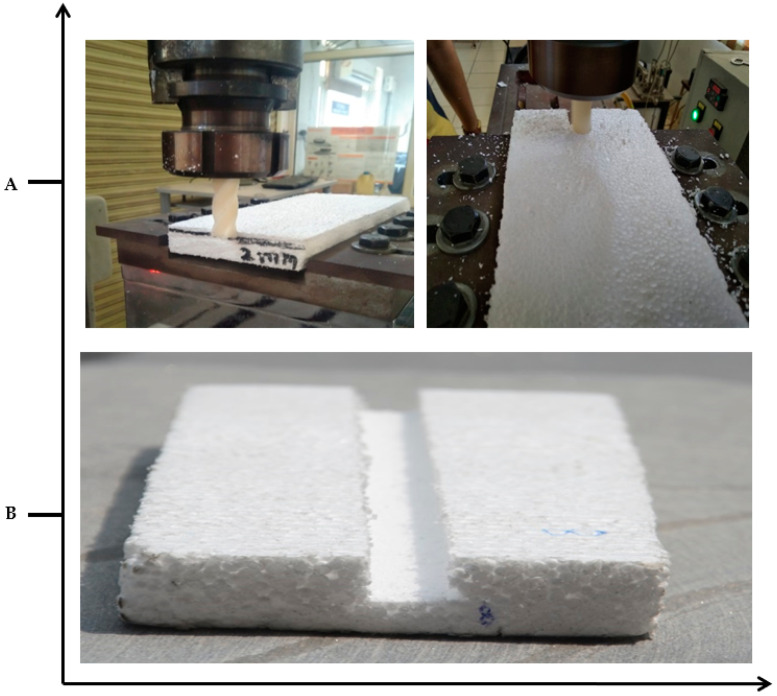
Pictorial views of the end-milling of the expandable polystyrene (EPS) using: A, 3D printed tool and B, as-machined EPS.

The milling machine available in the laboratory was utilized for the end milling of EPS with some modifications. The input process parameters and their levels are tabulated in Table 2. The process parameters were selected based on evidence available from the literature for surface roughness and dimensional accuracy of the end-milling operations [54,55]. Table 3 depicts the L9 standard array (the Taguchi’s design of experiments) utilized to conduct the test runs. Additionally, Figure 2 shows the pictorial representations of the end-milling of the EPS utilizing the 3D printed ABS tool, which finally produced a groove. The dimensional accuracy (DA) of the subsequently machined grooves was recorded by using a coordinate measuring machine (CMM) made by Accurate Spectra, Pune, India. Two-dimensional deviations were recorded in terms of the width and depth of the cut. The raw data in this regard was obtained by subtracting the change in dimension from the set dimension and named as deviation-in-width (DIW) and deviation-in-height (DIH). In the case of DIW, DA was recorded by subtracting the size measured with CMM and the diameter of the end tool. Whereas, in the case of DID, the raw data was obtained by subtracting the cut’s depth identified with CMM and the experimental value of the depth of cut (DoC).

The EPS is a soft material, and the measurement of the surface roughness with a universal stylus-based instrument might not be able to produce reliable outcomes. Therefore, in this work, a non-contact 3D optical profiler system (accuracy: 0.15 nm; make: NanoMap, NanoMap, AEP Technology, Santa Clara, CA, USA, 1000 WLI) was used to provide the average roughness value of the machined surface. Moreover, a Dino-lite microscope was utilized to capture the surface morphology of the machined parts. The microscope was calibrated by using a standard scale provided by the manufacturer. Images were captured at two different magnifications: 60× and 75×, to analyze the different machining parameters’ effects on the surface morphology. The Zeta Instruments Profilometer was utilized to record the optical profiles of the machined surfaces. 

## 3. Results and Discussion

### 3.1. Single Objective-Optimization

**Figure 3:**
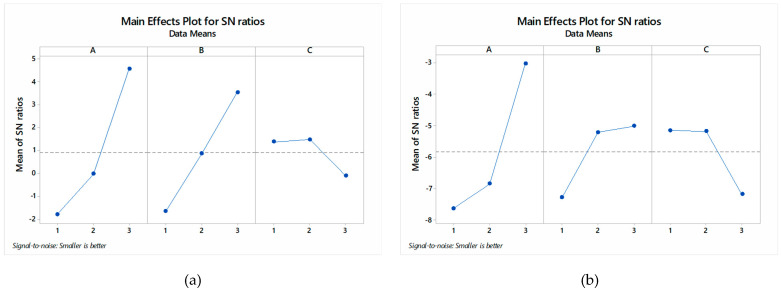

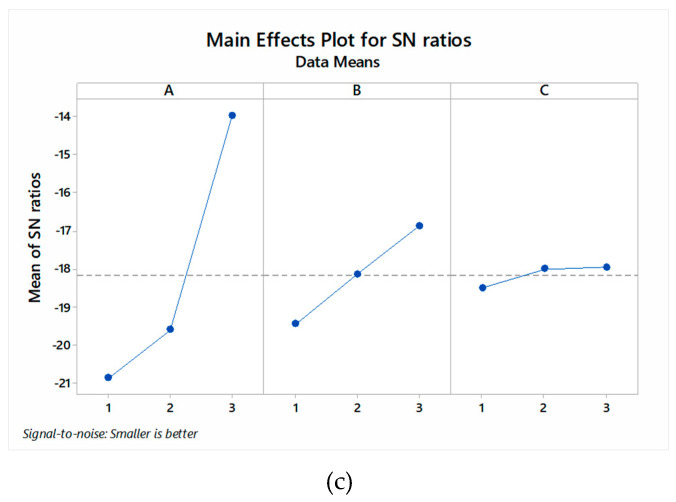
S/N ratio plots for DIW (**a**), DIH (**b**), and R_a_ (**c**). Note: A, B, and C represent feed rate, spindle speed, and depth-of-cut, respectively.

The recorded observations for DA and surface roughness (R_a_) are shown in Table 4, along with signal-to-noise (S/N). The responses, such as DIW, DIH, and R_a_, are “the smaller, the better” options and are optimized by using the Minitab-17 statistical software package. For a detailed description of the conversion of the raw data to the S/N ratio and plotting, the S/N ratio is well discussed elsewhere [56,57,58]. The main issue in this type of optimization is the difficulty in selecting the suitable input process parameters for all the considered outputs since the one optimized setting is likely to conflict with the others. On the other hand, this type of optimization is highly beneficial to get detailed insights into parametric effects as the observations are independent and free of influence from the different responses. The S/N ratio plots for DIW, DIH, and R_a_ are shown in Figure 3, to find the optimal parametric setting and effect of diverse variables. Figure 3 clearly depicts that in the case of DIW, the dimensional accuracy of the cut increases when the feed rate increases from 30 to 40 mm/min, and then further from 40 mm/min to 50 mm/min. This is mainly because, at a higher level of the feed rate, the time spent by the cutting tool in machining the groove was least. Therefore, the tool passed quickly across the cutting groove without causing thermal shocks to the work material. It was found that the EPS is highly thermal sensitive, and its structure consists of micro-balls that tend to squeeze upon thermal stimulus.

**Figure 4:**
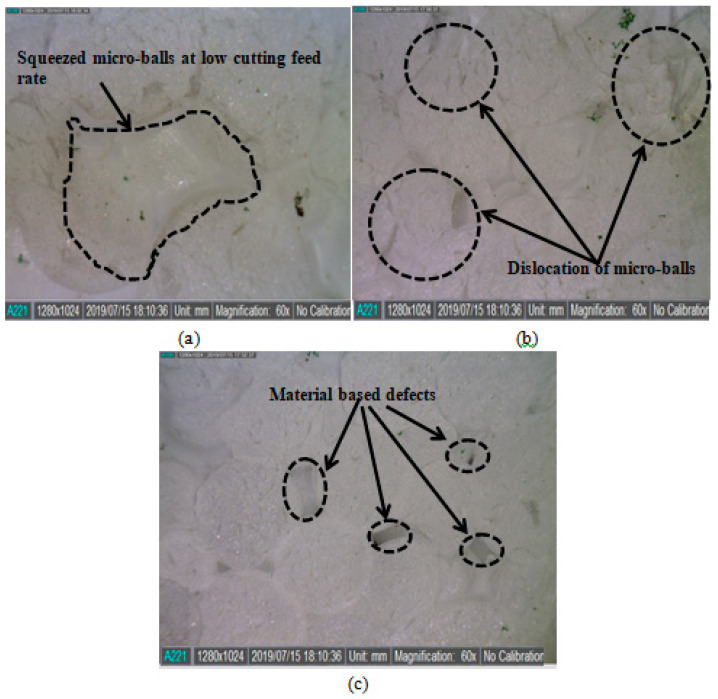
Surface morphology of (**a**) thermal squeezed (at low 30 mm), (**b**) dislocated (at low 30 mm), and **(c)** precisely machined micro-balls (at 50 mm/min).

The feed rate of the end-mill operation, in the present case, defines the magnitude of machining heat propagating through the work material. Therefore, the fast pass of the end-mill tool secures the work material from in-process heat production. As a result, the DIW feature of the as-machined was improved by increasing the feed rate to 50 mm/min. It is worth mentioning that interconnected micro-balls of EPS possess less bond strength. Hence, a low feed rate of the end-mill tool can cause dimensional deviations. Figure 4 shows (a) the effect of in-process thermal-stimulus originated squeezing and (b) dislocation of the micro-balls on the morphology of machined-EPS, at low feed rate. Whereas, Figure 4c shows the machined surface at 50 mm/min with no visible machining incurred surface defects. In the case of spindle speed, it can be seen from Figure 3 that the DIW is improved by increasing the spindle speed. The spindle speed in machining represents the cutting force applied to the work material. While machining EPS, it was observed that as the spindle speed was increased, the end-milling tool exerted greater force on the work material and imparted the brittle fractures. That usually happens in a fraction of seconds and leaves no room for the work material to produce abrupt cutting behaviors, for example pulling or dislocation of the micro-balls. 

**Figure 5:**
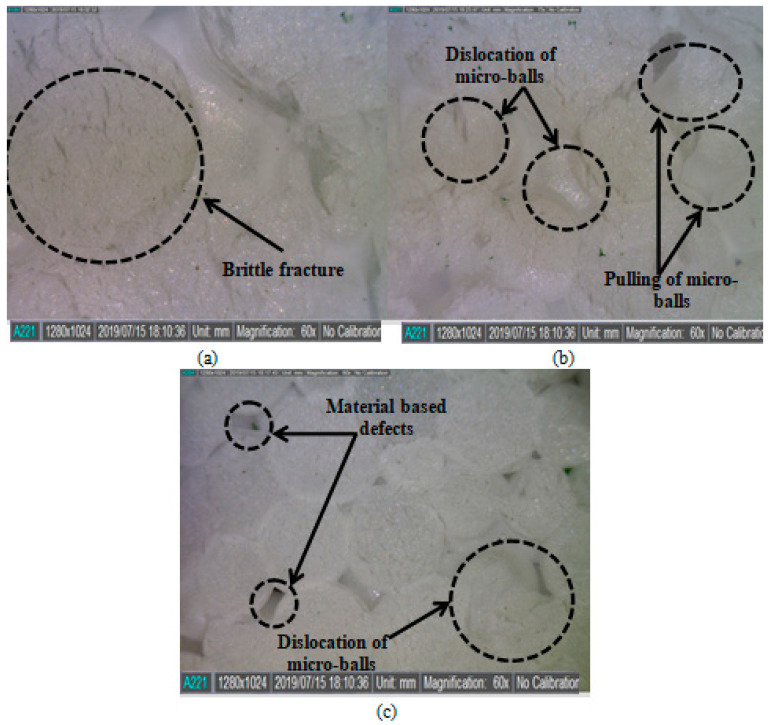
Surface morphology of machined EPS at (**a**) 1500 rpm, (**b**) 1700 rpm, and (**c**) 2000 rpm.

**Figure 6:**
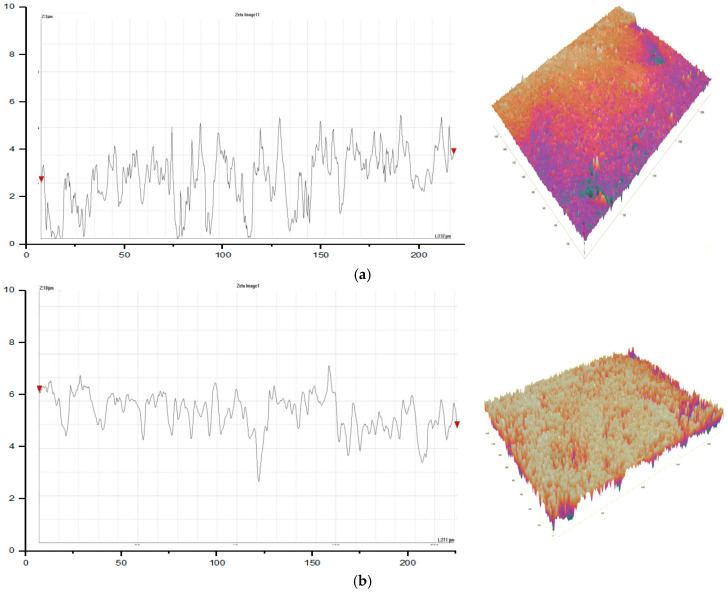

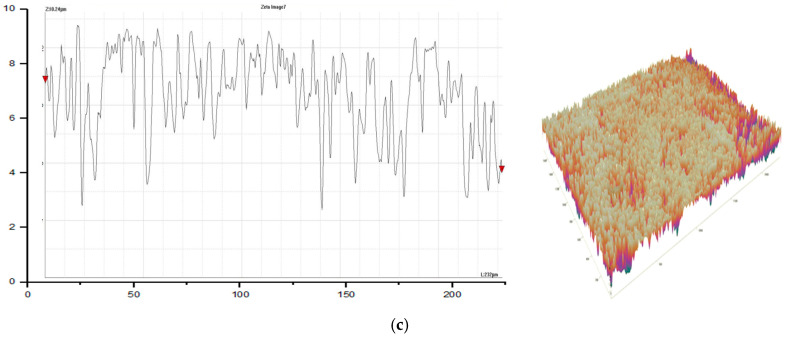
Surface profile and rendered surface profile: (**a**) 50 mm/min: R_a_ ~3.047 µm; (**b**) 40 mm/min: R_a_ ~6.633 µm, and (**c**) 30 mm/min.: R_a_ ~9.827 µm.

From Figure 5, the effect of spindle speed on the DIW can be characterized. It can be seen that at 1500 rpm of spindle speed, micro-ball dislocation was observed along with the brittle fracture; refer to Figure 5a. However, when the spindle speed was increased to 1700 rpm, the brittle fracture effect was eliminated. The localized micro-ball dislocation can still be seen; refer to Figure 5b. Finally, in the case of 2000 rpm spindle speed, comparatively less dislocation of the micro-balls was observed. Since the spindle speed has affected the surface morphology of the machined EPS work material; therefore, it has influenced the dimensional accuracy. Further, in the case of depth-of-cut, it was found that the DIW of the machined EPS did not affect an increase in the cut depth from 2 to 4 mm. However, with a further addition to 6 mm, the DIW reduced. This might be because when the cut depth was 6 mm, the machined micro-balls chips were stuck within the groove and resulted in the tool tightening within the groove. In the case of DIH, it is observed from Figure 3 that the effect of input process parameters is almost similar to the DIW. This indicates that the input process parameters induced similar effects on the observed DA, both DIW and DIH. The optimized process parameters for DIW and DIH are a feed rate of 50 mm/min, a spindle speed of 2000 rpm and a depth of cut of 2 mm. Further, in the case of R_a_, it is seen in Figure 3 that with an increase in the feed rate of the end-mill tool, the resulting R_a_ value decreases. Thus, the formation of surface unevenness is usual. The reason is similar, at low feed rate abrupt micro-ball dislocation, as well as their squeezing takes place. Further, similar effects were seen in the case of spindle speed. This means that for obtaining a finely finished machine surface, higher values of the spindle speeds are desirable. From the rendered and surface profile, refer to Figure 6: the machined surface at a feed rate of 50 mm/min, possesses surface roughness ~3.04 µm, whereas, in the case of 30 mm/min, the roughness is about three times higher, ~9.83 µm and at low feed rate, surface irregularities are higher. 

Moreover, From Table 5, it has been found that the percentage contribution of residual error in the case of DIW, DIH, and R_a_ is 2.89%, 2.43%, and 1.10%, respectively. Residual errors are lower than 5% for all responses, indicating the recording of data below the acceptable level of error. Table 6 shows the response values of the S/N ratio for the three different levels of input parameters. The values of Table 6 were used for predicting the optimized S/N ratio (β_opt_) for DIW, DIH, and R_a_ by using Equation (1):

β_opt_ = ḿ + (ḿ_1max_ − ḿ) + (ḿ_2max_ − ḿ) + (ḿ_3max_ − ḿ), (1)

where, ḿ is the overall mean of the S/N ratio (refer to Table 4), ḿ_1max_, ḿ_2max_, and ḿ_3max_ are maximum S/N ratio for 1st input parameter, 2nd input parameter, and 3rd input parameter, respectively. The values ḿ_1max_, ḿ_2max_, and ḿ_3max_, correspond to Table 6.

The obtained β_opt_ for DIW, DIH, and R_a_ is given in Equations (2)–(4):

β_opt_ = 0.904 + (4.53868 − 0.904) + (3.52936 − 0.904) + (1.46935 − 0.904) = 7.729 db, (2)

β_opt_ = -5.833 + (−3.029 + 5.833) + (−5.011 + 5.833) + (−5.311 + 5.833) = −1.585 db, (3)

β_opt_ = -18.15 + (−13.97 + 18.15) + (−16.87 + 18.15) + (−17.86 + 18.15) = −12.4 db. (4)

Equation (5) shows the formula to evaluate the output response from the predicted response S/N ratio and is utilized in the present work to compute responses.

Y_opt_^2^ = 1/10^βopt/10^, (5)

where Y_opt_ is the optimal response. Furthermore, to validate the accuracy of predicting output responses, confirmation experiments (n = 3) were also performed at the suggested optimized parametric setting. Table 7 depicts the expected and confirmatory experiment results for the output responses. The predicted and confirmatory result shows good correlation and, therefore, validates the statistical analysis.

### 3.2. Tool Performance

**Figure 7:**
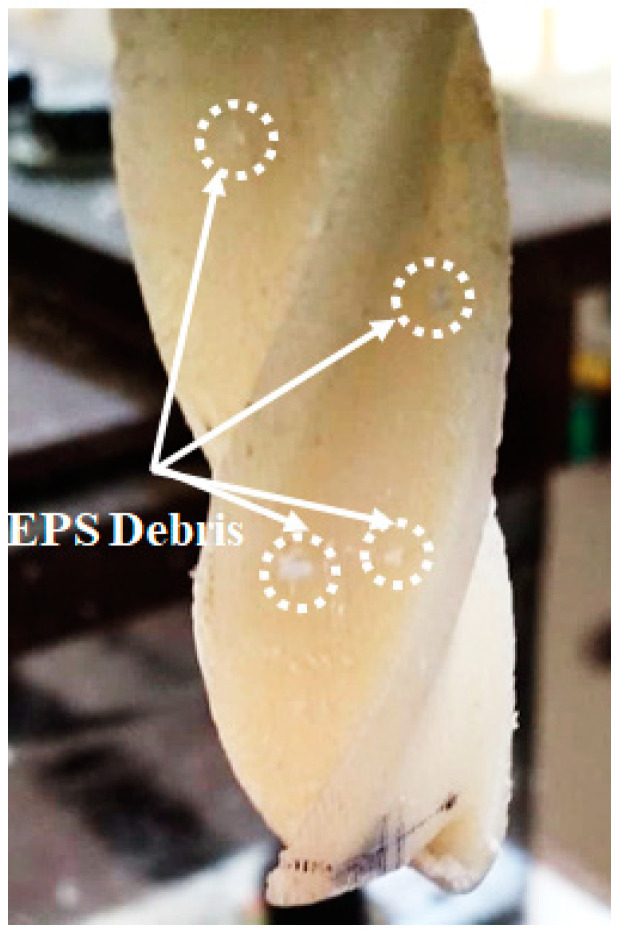
Pictorial view of the deposition of EPS debris on 3D printed ABS tool.

The performance of the 3D printed tool was assessed by recording the tool wear rate. For this, the initial and final weight of the tool was measured using a digital weighing scale (accuracy 0.001 mg). It was found that despite losing weight after machining, for all the EPS samples, no weight loss was recorded. Instead of weight loss, the 3D printed ABS tool gained weight of about 0.002 mg, because of the adhesion of the EPS machine chip on the printed tool. The material adhesion was mainly because of the heat produced at the work material, and at the 3D printed ABS tool. Figure 7 shows the pictorial view of the EPS’ debris deposit on the tool. Furthermore, it was found that the ABS tool was free of any type of crack, distortion, or worn cutting edges. It was found that the 3D printed based thermoplastic machining tools are efficient in obtaining desirable quality characteristics in case of soft polymeric materials. Moreover, the wear resistance of the developed tools enables machining the soft polymers for batch and mass production runs. 

The novel applications of the 3D printing technologies to develop different types of machining tools will not only help to improve the machining efficiencies but also enable cutting-down the production times and tooling costs. In contrast, the metallic and ceramic-based 3D printing technologies can utilize harder feedstock systems to develop a cutting tool for machining different types of engineering materials. The polymer-based 3D printing technologies can use the feasibility of using ceramic or metallic reinforcements for enhancing the hardness and wear resistance of the cutting tools to the next possible level for cutting harder polymers. There have been many examples [32,33,34,35] where the reinforced 3D prints have attained improved mechanical, thermal, and wear-resistant properties. However, the potential of such feedstock systems to be used for machining polymeric materials, as well as micro-machining of comparatively harder metals and ceramic, must be explored.

In the present study, the EPS was used as the work material that exhibits much less hardness (~65 RM) and poor interatomic bonding of the pre-expanded polystyrene beads, therefore, during machining thermal shock was not seen. Furthermore, the low thermal conductivity of the EPS, ~0.034 W/mK, provided the inherent thermal-insulation to avoid any damages caused by the heat concentration during machining. However, in case of other polymeric work materials, it is essential to record the thermal images at the tool-workpiece interface in order to generate supportive knowledge.

## 4. Conclusions

In the current study, a novel application of a 3D printed ABS end-mill tool was explored for machining soft polymer (EPS). Based on geometrical and surface characteristics of a machined EPS, the following conclusions can be drawn.

Through scrutiny, it was found that the dimensional accuracy (of both DIW and DIH) and surface finish of the machined EPS can be improved by increasing the feed rate. From the surface morphology of the machined EPS, it was found that by increasing the feed rate machining issues of EPS, such as thermal sensitivity and material dislocation can be controlled. Furthermore, it was found that at a higher level of spindle speed, the dimensional accuracy and surface finish of the machined EPS improved. This is because, at a lower level of the spindle speed, the cutting mechanism included micro-balls dislocation followed by brittle fracture. However, in the case of depth-of-cut, the optimized level corresponding to dimensional accuracy and surface finish is 2 mm. The optimized process parametric levels, in regard to dimensional accuracy and surface roughness, have been obtained and verified statically through ANOVA. It was found that the feed rate is the only statistically significant process parameter for all output responses. Along with this, the predicted optimized parametric levels and S/N ratios were verified through confirmatory experiments, where a strong correlation was found between the predicted and experimental values.

The performance analysis of the 3D printed ABS tool highlighted that the developed tool is capable of machining soft polymers with controlled dimensional and topographic features. Furthermore, investigations should explore the potential of the reinforced 3D printed tool for machining tough and harder polymers.

## Figures and Tables

**Table 1:** List of machining activities on soft polymers.

Tool Material	Machine	Material Cutting	Summary
Stainless Steel	CNC Milling Machine	Polyurethane	The researchers found that surface roughness of tested samples was significantly affected by cell size and depth-of-cut. The additive manufacturing-based tooling provided a good alternative to conventional CNC-based tooling based on its low cost and rapid turnover [48].
Hot-Wire Frame Cutter	Flexible Automated System (FAS)	Polyurethane foams	The FAS significantly reduced the cutting time and improved cutting quality. It has more flexibility to handle dissimilar geometries and more advantages over molding while making foam cushions [49].
Hot Wire, Water Jet, Hot Ribbon Hot Tool	Free Form Automated Sculpting Technology, True Surface System, Shape Maker, Model Angelo, Free Foam Thick-Layered Object Manufacturing, Variable Lamination Manufacturing, Rapid-Heat Ablation, Michelangelo	Polyurethane foams	The review paper described a different kind of cutting machine form. Suggested and discussed the relative merits of rapid prototype systems to enhance foam cutting systems [50].
High-Speed Steel	Milling	Flexible polyurethane foam	In this work, it was observed that at spindle speed ~2400 rpm and feed rate ~2400–4000 mm/min is suitable for distortion-less geometries. It is likely to build up customized products to convince the explicit requirements of persons with disabilities [51].
Hot Wire	Four Axis Automated Hotwire Cutter	Polyurethane foam	The part quality and dimensional accuracy depend on machining parameters. The work investigated the part quality and dimensional accuracy while hot cutting of foam in two different cutting angles and found that cutting parameters influenced the quality of the parts [52].
Drill, Mill Cutter, Saw Cutter	Lathe, Milling, Sawing	Polyurethane foam	The work presented an excellent application of the process for orthopedics [53].

**Table 2:** Selected input process parameters and their levels.

Parameters	Feed Rate, F	Spindle Speed, S	Depth of Cut, DoC
Units	mm/min	rpm	mm
S. No.	A	B	C
Level 1	30	1500	2
Level 2	40	1700	4
Level 3	50	2000	6

**Table 3:** Experimentation approach based on the designed experimental log.

Exp. No.	A	B	C
1	30	1500	2
2	30	1700	4
3	30	2000	6
4	40	1500	4
5	40	1700	6
6	40	2000	2
7	50	1500	6
8	50	1700	2
9	50	2000	4

**Table 4:** Observed results for deviation-in-width (DIW), deviation-in-height (DIH), and surface roughness (Ra).

S. No.	Deviation-In- Width, DIW (mm)	S/N Ratio (dB)	Deviation-In- Height, DIH (mm)	S/N Ratio (dB)	Surface Roughness, R_a_ (µm)	S/N Ratio (dB)
1	1.45	−3.227	2.55	−8.130	13.02	−22.2982
2	1.27	−2.076	2.21	−6.887	11.52	−21.2298
3	1.01	−0.086	2.47	−7.853	9.01	−19.0964
4	1.25	−1.938	2.33	−7.347	10.56	−20.4799
5	1.05	−0.423	2.32	−7.309	9.08	−19.1636
6	0.77	2.2702	1.97	−5.889	9.11	−19.1980
7	0.98	0.1755	2.08	−6.361	6.02	−15.5991
8	0.56	5.0362	1.18	−1.437	5.01	−14.0002
9	0.38	8.4043	1.16	−1.289	4.12	−12.3043
Overall S/N ratio, dB	-	0.904	-	−5.833	-	−18.15

**Table 5:** ANOVA results for DIW, DIH, and R_a_.

Source	Degree of Freedom	Sum of Square	Variance	Fisher’s Value	Probability (P)	Contribution (%)
DIW						
F	2	64.133	32.067	19.75	0.048 *	57.01
S	2	40.462	20.231	12.46	0.074	35.97
DoC	2	4.656	2.328	1.43	0.411	4.14
Residual Error	2	3.248	1.624			2.89
DIH						
F	2	36.302	18.1508	27.09	0.036 *	65.77
S	2	9.465	4.7325	7.06	0.124	17.15
DoC	2	8.092	4.0459	6.04	0.142	14.66
Residual Error	2	1.34	0.6699			2.43
R_a_						
F	2	60.754	40.5863	30.37	0.013 *	84.01
S	2	9.074	5.0431	4.536	0.081	12.56
DoC	2	1.613	0.6195	0.806	0.331	2.24
Residual Error	2	0.7971	0.6699			1.10

Note: * Indicates statistically significant variables.

**Table 6:** Delta rank of S/N responses.

Level	F	V	DoC
**DIW**			
1	−1.79662	−1.66336	1.35969
2	−0.03060	0.84546	1.46335 *
3	4.53868 *	3.52936 *	−0.11158
Delta	6.33530	5.19272	1.57493
Rank	1	2	3
**DIH**			
1	−7.624	−7.280	−5.153 *
2	−6.849	−5.212	−5.175
3	−3.029 *	−5.011 *	−7.175
Delta	4.595	2.269	2.022
Rank	1	2	3
**R_a_**			
1	−20.87	−16.87 *	−17.92
2	−19.61	−18.13	−17.86 *
3	−13.97 *	−19.46	−18.68
Delta	6.91	2.59	0.82
Rank	1	2	3

Note: * indicates a maximum S/N ratio.

**Table 7:** Statistically predicted and confirmatory experimental values for output responses.

Output Response	Predicted	Experimental	Deviation (±)
DIW (mm)	0.410	0.415	0.05
DIH (mm)	1.018	1.107	0.089
R_a_ (µm)	4.16	4.11	0.05
